# Fine‐root dynamics in deeper soils: a critical but overlooked component of ecosystem responses to climate warming

**DOI:** 10.1111/nph.70326

**Published:** 2025-06-25

**Authors:** Steve Kwatcho Kengdo, M. Luke McCormack, Ivika Ostonen, Margaret S. Torn

**Affiliations:** ^1^ Earth and Environmental Sciences Area Lawrence Berkeley National Laboratory Berkeley CA 94720 USA; ^2^ The Center for Tree Science The Morton Arboretum Lisle IL 60532 USA; ^3^ Institute of Ecology and Earth Sciences University of Tartu 50409 Tartu Estonia

**Keywords:** climate warming, fine‐root biomass, fine‐root dynamics, root exudates, soil organic carbon cycling

## Abstract

Climate warming is predicted to strongly affect the functioning of terrestrial ecosystems. The plant root system is a critical component of these ecosystems, with fine roots, in particular, playing a key role in plant water and nutrient uptake and transport. In addition, root litter and exudation represent the dominant plant carbon inputs into soil. Consequently, understanding fine‐root responses to warming is essential for predicting how the growth, resilience, and carbon storage of terrestrial ecosystems will respond to future climate change. Despite the growing literature on fine‐root responses to warming, most studies have focused on topsoil (0–30 cm). However, a significant portion of the fine‐root mass occurs below this depth. For instance, *c.* 40% of fine‐root mass is found below 30 cm in temperate and tropical ecosystems. Due to the importance of fine roots for plants and belowground carbon cycling, focusing solely on surface soils overlooks the critical need for insights into how roots in deeper soil layers (e.g. below 30 cm) respond to warming. We argue that studying the entire soil profile is necessary to comprehensively understand fine‐root dynamics under climate warming and the implications for soil organic carbon cycling, water, and nutrient uptake.

## Fine roots as drivers of carbon cycling

**Fig. 1:**
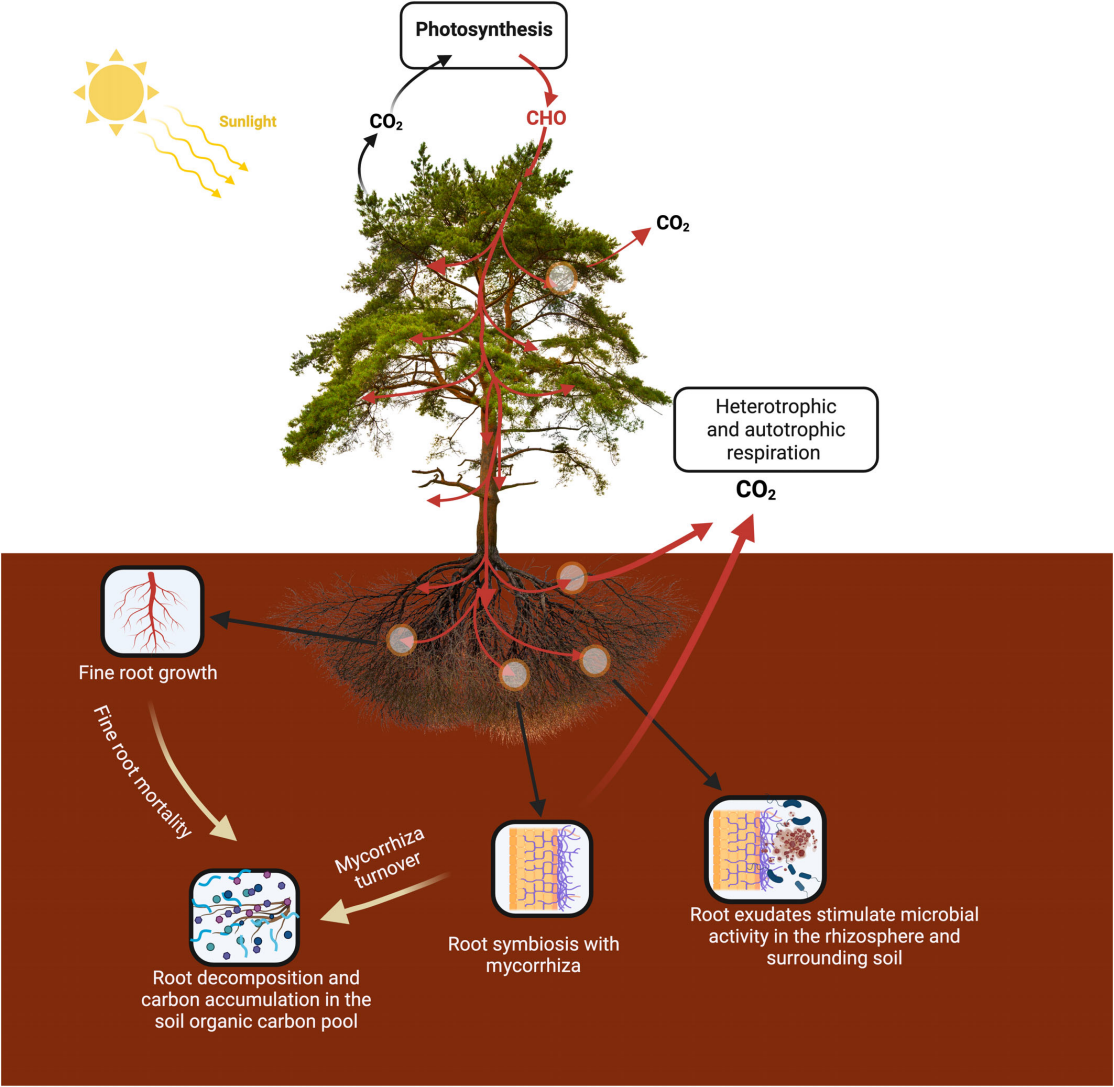
Conceptual illustration of how fine roots influence soil organic carbon. Created in BioRender. Kwatcho Kengdo, S. (2025) https://BioRender.com/5nrmrjx.

Fine roots (diameter < 2 mm) account for roughly 22% of global terrestrial primary productivity (McCormack *et al*., 2015) and play a crucial role in soil carbon cycling. They influence carbon cycling through a range of processes, including root turnover and litter input, root exudation and rhizosphere priming effects on soil organic matter (SOM) decomposition, root respiration, and symbiotic relationships with mycorrhizal fungi and bacteria (Fig. 1; Hopkins *et al*., 2013; Keiluweit *et al*., 2015; Pausch & Kuzyakov, 2018; Solly *et al*., 2018; Chari & Taylor, 2022). Beyond their functions in water and nutrient uptake, fine roots serve as a key input of organic carbon into soils (Rasse *et al*., 2005). A significant proportion of photosynthetically fixed carbon (C) is allocated belowground to support root production and maintenance. Upon root senescence or mortality, root detritus decomposes, with C either being stabilized into Soil Organic Carbon (SOC) pools or respired back to the atmosphere. Through these interconnected processes, carbon fixation, allocation, root production, mortality, and turnover, fine roots are important components of soil carbon cycling and significantly influence SOC dynamics (Pregitzer, 2002; Brunner & Godbold, 2007; Keller *et al*., 2021). Root exudation, the release of low‐molecular‐weight organic compounds (i.e. exudates) into the surrounding soil, regulates SOC cycling in the rhizosphere (Heinzle *et al*., 2023). Root exudates are a primary energy source for soil microbes and can alter the size and sink strength of the SOC pool (Kuzyakov *et al*., 2000; Meier *et al*., 2017). They stimulate soil microbial activity, leading to increased decomposition of SOM. Additionally, root exudates may contribute to SOM turnover through direct enzymatic degradation of organic compounds and promote soil aggregate formation, affecting SOC stabilization (Weisskopf *et al*., 2006; Baumert *et al*., 2018). Fine‐root interactions with ectomycorrhizal (EcM) fungi further influence carbon cycling. These fungi extend the root's functional surface area, enhancing nutrient and water uptake while also affecting soil respiration (Heinemeyer *et al*., 2007; Wallander *et al*., 2013). Ectomycorrhizal fungi contribute to SOC dynamics through microbial necromass turnover (Klink *et al*., 2022; Camenzind *et al*., 2023). Certain lineages of EcM fungi may produce enzymes involved in SOM degradation, although the extent of this process remains unclear (Shah *et al*., 2016; Pellitier & Zak, 2018; Zak *et al*., 2019). Ectomycorrhizal fungi can also suppress SOM decomposition by releasing antifungal compounds that inhibit decomposer microbial communities; these effects can be large enough to alter the balance between C sequestration and loss (Keller *et al*., 2005). Soil respiration, the largest terrestrial C flux, integrates heterotrophic respiration (from microbial respiration and decomposition of SOM) and autotrophic respiration (from plant roots). Globally, autotrophic respiration accounts for *c*. 40% of total soil respiration (Schlesinger & Andrews, 2000; Friedlingstein *et al*., 2022; Jian *et al*., 2022), making it an important source of ecosystem carbon fluxes.

Climate warming is projected to stimulate plant growth and increase belowground net primary production across various ecosystems and soil depths (Wang *et al*., 2021). Rising temperatures may subsequently affect all the aforementioned processes. For instance, greater fine‐root biomass can elevate root exudation and litter input and expand the rhizosphere zone. Moreover, temperature‐driven changes in root respiration may contribute to higher CO_2_ fluxes. Shifts in fine‐root morphology and mycorrhizal associations under warming could affect nutrient and water uptake while influencing nutrient release from the decomposition of organic matter (Dawes *et al*., 2017; Solly *et al*., 2017). These root‐mediated responses to warming may collectively reshape carbon, water, and nutrient cycling, thereby altering long‐term ecosystem function and stability (Raza *et al*., 2025). However, the magnitude and nature of these effects may differ substantially between shallow and deep soil layers.

## Fine‐root responses to warming in the topsoil

**Fig. 2:**
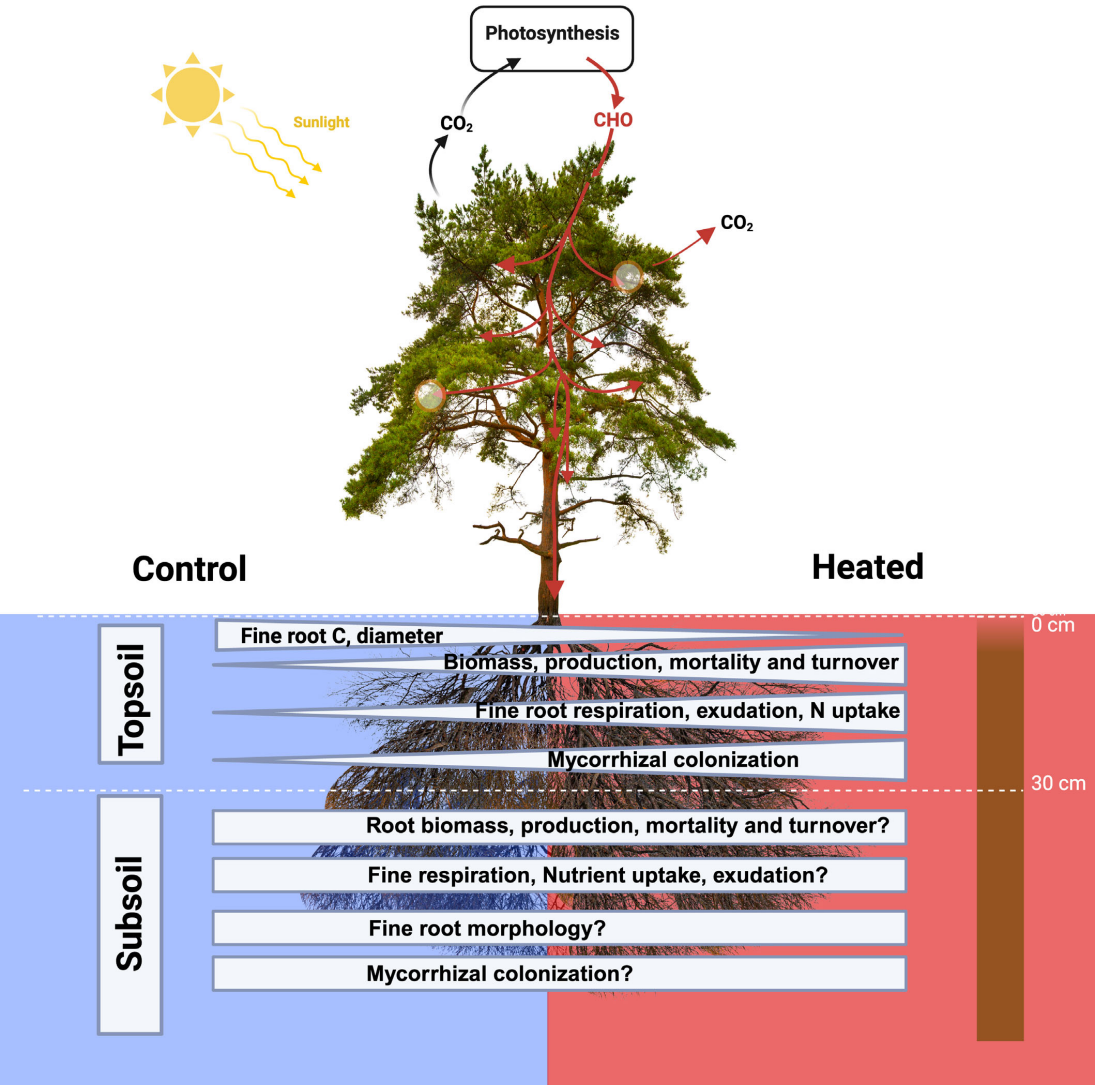
Schematic representation of the observed effects of warming on fine roots in topsoil (0–30 cm) and the poorly tested responses in subsoil (below 30 cm). Question marks indicate that the response in deep soils is highly uncertain. Created in BioRender. Kwatcho Kengdo, S. (2025) https://BioRender.com/5nrmrjx.

Building on the mechanisms described above, we now explore how fine roots respond to warming. Research on fine‐root responses to warming has primarily focused on the topsoil, likely due to its accessibility and the high root density in shallow soil layers, where at least 50% of the total fine‐root system is typically found (Jackson *et al*., 1996; Schenk & Jackson, 2002; Brassard *et al*., 2009). This emphasis also stems from the assumption that changes in fine‐root mass and functional traits in the topsoil are more relevant to overall ecosystem functioning than those in deeper layers (Weemstra *et al*., 2023). Studies have shown that warming affects topsoil roots (Supporting Information Table S1) by altering fine‐root biomass, morphology, exudation, respiration, chemistry, and mycorrhizal associations (Wan *et al*., 2004; Björk *et al*., 2007; Zhou *et al*., 2011; Leppälammi‐Kujansuu *et al*., 2013; Parts *et al*., 2019; Malhotra *et al*., 2020; Wang *et al*., 2021; Kwatcho Kengdo *et al*., 2022, 2023; Heinzle *et al*., 2023; Liu *et al*., 2024a,b). These results suggest we could also expect significant effects on fine roots below 30 cm, but less is known about these effects (Fig. 2). Because fine roots grow from the same plants and share the same carbon and nutrient fluxes, the shallow and deep fine‐root system functions as a physiological and functional continuum (Ostonen *et al*., 2017), an interconnected network rather than independent and discrete entities. As a result, the warming effects observed in surface soils may either propagate to deeper soil layers, or root systems may adjust carbon allocation between top and subsoil, resulting in inconsistent responses among soil layers, depending on environmental conditions and the availability of nutrients (Stocker *et al*., 2023). The emphasis on topsoil, therefore, overlooks potential fine‐root responses in deeper soil layers, which may differ qualitatively or quantitatively.

## The overlooked fine‐root responses in subsoil and implications for soil organic carbon and nutrient cycling

Approximately 40% of fine‐root mass may reside below 30 cm in many ecosystems (Jackson *et al*., 1996), with some ecosystems displaying a secondary peak of fine‐root mass below a meter in depth (Lu *et al*., 2022). In some systems, deep rooting is an adaptive response to resource limitation. For example, in cold, dry, or rocky environments, plants may develop specialized root morphology and architecture to acquire water and mineral‐derived nutrients, such as phosphorus, that are scarce in the topsoil due to rapid uptake and competition but are more available in deeper soil layers through mineral weathering. Therefore, subsoil fine roots are crucial for plant survival and nutrient cycling, affecting how ecosystems respond to environmental changes as a whole.

The subsoil, that is soils below 30 cm soil depth, contains the majority of total SOC (Jobbágy & Jackson, 2000), and is often characterized by lower turnover times compared to topsoil carbon, with estimated mean residence times of up to several thousand years (Rumpel & Kögel‐Knabner, 2011). The subsoil also typically has lower and more stable temperatures, low oxygen concentration, lower rooting densities, thicker and less branched roots, and lower concentrations of root‐derived carbon input (dead roots, rhizodeposition) than surface layers (Germon *et al*., 2020; Button *et al*., 2022). With increasing soil depth, root carbon inputs via rhizodeposition and extracellular substances become the primary contributors to SOM formation and soil aggregation (Wang *et al*., 2014; Baumert *et al*., 2018).

Although not widely recognized, deep soil layers will experience the full magnitude of climate warming. Models from the Climate Model Intercomparison Project 5 predict that subsoil will warm roughly at the same rate as topsoil (Soong *et al*., 2020). Furthermore, experimental evidence highlights that whole soil warming by +4°C increased soil CO_2_ efflux by 30–35% at all depths to 1 m (Hicks Pries *et al*., 2017), and heated plots had 33% less subsoil carbon (Soong *et al*., 2021) than did control plots, primarily due to having less plant‐derived organic matter.

Recent meta‐analyses (e.g. Wang *et al*., 2021) indicate that experimental warming significantly increases fine‐root biomass, with the most pronounced effects observed in deeper layers than in the topsoil, possibly due to increased water stress from warming. This result, however, is mainly based on studies in temperate and grassland ecosystems, with limited data from tropical or boreal regions. Increased root activity in deeper soil layers with warming implies more root litter and rhizodeposits (root exudates, secretion, cell senescence), which may enhance the quantity of labile carbon inputs in deeper layers (Rasse *et al*., 2005). This, in turn, would likely stimulate the growth and activity of saprotrophic microbes and mycorrhizal fungi, both of which could lead to a priming effect that accelerates litter and SOM mineralization (Baumert *et al*., 2018). Notably, the increased biomass production with warming observed in surface soils may be counterbalanced by increased mineralization of litter and SOM in subsoils. However, the net effect on deep SOC will depend on the balance between these processes and the relative rates of carbon stabilization vs loss (Bradford *et al*., 2016). Recent findings further suggest that warming can adversely affect microbial biomass carbon by stimulating its production and accelerating its decomposition (Liu *et al*., 2024a,b), underscoring the potential for soil warming to increase carbon losses and weaken microbial pathways for carbon stabilization, even when root input increases.

Altered dynamics of fine roots in the subsoil would also affect plant nutrients and water cycling. Increasing belowground allocation and root production in the subsoil would increase access to nutrient and groundwater resources. This could, therefore, promote plant growth and improve overall plant resilience. Thus, overlooking the response of the entire fine‐root system to warming gives an incomplete understanding of plant adaptation, plant functional trait responses, and ecosystem responses to warming. In addition, many Earth system models do not have depth‐resolved soil biogeochemistry, and most are parameterized with data collected only from topsoils. This complicates predictions of future ecosystem responses to warming and may lead to an underestimation of the role of deep fine roots in mitigating or amplifying the effects of warming in the subsoil. This introduced uncertainties in future climate projections. Furthermore, global change factors often co‐occur; the interactions between deep fine‐root responses to warming and altered precipitation, CO_2_ fertilization, and elevated nitrogen may further create complex fine‐root responses with their related ecosystem impacts.

## Methodological challenges and future directions

Observing and capturing the dynamics of fine roots across the entire soil profile remains challenging due to methodological constraints. Root mass density (root mass per unit soil volume) typically declines with soil depth, and high spatial heterogeneity can pose challenges in detecting significant changes and trends in the subsoil, necessitating greater sample numbers. Soil compaction also makes it difficult to sample and access intact roots in deeper soil layers. Sparsity and compaction impact the installation and analysis of root‐observation windows such as minirhizotrons. Moreover, few experiments have been able to manipulate the deeper soil environment, leading to significant knowledge gaps in how warming, nutrient cycling, and plant–soil interactions change at depth. All these factors may exacerbate the logistical and cost challenges of sampling roots.

These challenges highlight the need for both innovative and standardized methodological approaches to study fine roots in subsoils. First of all, depth‐resolved sampling and imaging techniques are required, likely relying on traditional coring tools but encompassing deeper soil depths. Standardizing protocols for sampling fine roots from deep soil layers will enhance comparability across studies and sites. High‐resolution imaging, particularly using approaches such as minirhizotrons, should be deployed to enable continuous monitoring of root growth, turnover, and interactions with mycorrhizal fungi. While noninvasive technologies, such as ground‐penetrating radar, offer potential for detecting coarse roots (Stover *et al*., 2007), their application to fine roots and deeper roots in general remains limited.

In addition, manipulative and controlled approaches such as Ecotrons and weighing lysimeters will help simulate the effects of warming and provide insights into root responses to climate warming (Castanha *et al*., 2018). Isotopic labeling techniques (^11^C, ^13^C, ^15^N, ^18^O) coupled with mass spectrometry imaging can be used to track short‐ or long‐term carbon and nutrient allocation to deep fine roots, building on shallow‐root studies (Epron *et al*., 2012) and their interactions with soil microbial communities and organic matter.

The integration of seasonal and long‐term responses should be considered. Because climate change is altering freeze and thaw cycles, winter warming experiments should help examine how frost and warming interact to influence root vertical distribution, growth, and microbial activity at depth, for example. This is highly relevant for permafrost soils, where plants generally have shallow root systems and where permafrost thaw could have cascading effects not only on plants and root growth but also on soil carbon and nitrogen dynamics (Blume‐Werry *et al*., 2019). Long‐term warming manipulations not only in temperate zones but also in underrepresented ecosystems, such as tropical and boreal ecosystems, are required to capture the short‐ and long‐term responses of fine roots across the complete soil profile.

The priming effect deserves particular attention. Since root exudation can either accelerate SOC loss or enhance SOC stabilization (Chari & Taylor, 2022; Brunn *et al*., 2025), studies are needed that focus on measuring root‐derived carbon fluxes to distinguish between new and old carbon sources across the soil profile and to identify the microbial communities involved in deep SOC dynamics using stable isotope probing (Hungate *et al*., 2015).

As noted above, fine roots do not function in isolation. Instead, they form an intricate physical and functional continuum with mycorrhizal fungi. Mycorrhizal communities, and likely their function, shift within the soil profile (Lindahl *et al*., 2007; Mucha *et al*., 2024). However, these shifts have been noted over relatively short profiles of *c*. 10 cm and very rarely deeper than *c*. 50 cm (Mucha *et al*., 2024). Given the paucity of studies characterizing mycorrhizal communities below 50 cm, it is yet unknown if and how deeper mycorrhizal communities systematically differ from communities in shallow soil; thus, little is known about their likely responses as deeper soils progressively warm. Still, results from warming studies in shallow soils suggest that community shifts associated with warming may be more significant than shifts related to other environmental changes, including nitrogen enrichment, and will lead to functional changes in the mycorrhizal community with potential decreases in overall diversity but also increases in the proportions of hydrolytic enzymes driving soil carbon loss (Anthony *et al*., 2021). Further research should integrate depth‐resolved mycorrhizal sampling and functional assays to determine how they change with soil depth and warming.

Finally, given the variation in fine‐root responses across ecosystems, global research networks are required to synthesize available data and improve predictive models. Therefore, collaboration across disciplines is essential to integrate physiological, ecological, and molecular responses of deep fine roots to warming. Initiatives such as the Deepsoil2100 network (Protti Sánchez *et al*., 2024) could build on existing efforts and foster research collaborations to explore how fine roots respond to climate warming throughout the whole soil profile.

## Conclusion

Fine roots in deeper soil layers remain underrepresented in experimental and modeling studies despite their role in carbon and nutrient cycling. ‘Digging deeper’ (Iversen, 2010) is crucial for understanding the mechanisms of plant and soil responses to climate warming and, therefore, developing accurate predictions of ecosystem responses. Overcoming methodological challenges through depth‐resolved sampling, technological advances, manipulative and long‐term warming experiments, and interdisciplinary collaboration will significantly improve our ability to predict how terrestrial ecosystems respond to global change.

By expanding our focus beyond surface soils, we can develop a more holistic understanding of belowground processes and refine climate models. While a significant proportion of fine‐root biomass is found below 30 cm in many ecosystems (Lu *et al*., 2022), we recognize that this pattern is not universal. For example, in boreal forests and tundra ecosystems, where permafrost and shallow active layers limit root penetration, fine‐root biomass is often concentrated in the upper 10–20 cm (He *et al*., 2023). By contrast, in temperate forests, grasslands, and tropical ecosystems, where soil depth and moisture availability allow for deeper rooting, fine roots can extend well below 30 cm. Given these differences, studies of deep fine‐root dynamics should be interpreted in the context of ecosystem‐specific constraints. However, site‐specific variability should not prevent broader generalization, and cross site comparisons and synthesis studies are encouraged.

## Competing interests

None declared.

## Author contributions

SKK wrote the first draft of the manuscript with contributions from MLM, IO and MST.

## Disclaimer

The New Phytologist Foundation remains neutral with regard to jurisdictional claims in maps and in any institutional affiliations.

## Supporting information


**Table S1** Summary of studies on fine‐root responses to warming.
Please note: Wiley is not responsible for the content or functionality of any Supporting Information supplied by the authors. Any queries (other than missing material) should be directed to the *New Phytologist* Central Office.
