# Supplementary material for: Fine‐root dynamics in deeper soils: a critical but overlooked component of ecosystem responses to climate warming
Source: New Phytol. 2025 Jun 25;247(6):2507–13. doi: 10.1111/nph.70326 (PMC12371150; doi:10.1111/nph.70326)
Supplement: Supplementary file 1 — Table S1 Summary of studies on fine‐root responses to warming. Please note: Wiley is not responsible for the content or functionality of any Supporting Information supplied by the authors. Any queries (other than missing material) should be directed to the New Phytologist Central Office. [file NPH-247-2507-s001.docx]

New Phytologist Supporting information

**Article title: Fine root dynamics in deeper soils: A critical but overlooked component of ecosystem responses to climate warming**

Authors: Steve Kwatcho Kengdo^1*^, M. Luke McCormack^2^, Ivika Ostonen^3^, Margaret S. Torn^1^

^1^ Earth and Environmental Sciences Area, Lawrence Berkeley National Laboratory, Berkeley, CA 94720, USA

^2^ The Center for Tree Science, The Morton Arboretum, Lisle, IL 60532, USA

^3^ Institute of Ecology and Earth Sciences, University of Tartu, 50409 Tartu, Estonia

Article acceptance date: 30 May 2025

The following Supporting Information is available for this article:

Table S1: Summary of Studies on Fine Root Responses to Warming: Covering Warming Intensity, Ecosystem Type, and affected traits including Fine Root Biomass (FRB), Necromass (FRN), Production (FRP), along with Morphological Traits like Specific Root Length (SRL), Specific Root Area (SRA), Root Tissue Density (RTD), and Average Diameter (AvgD).

| **Reference** | **Temperature Treatment (°C)** | **Warming Type** | **Soil Layer** | **Ecosystem** | **Trait(s) Affected** |
| --- | --- | --- | --- | --- | --- |
| Melillo et al. (2011); Zhou et al. (2011) | +5 | Soil | Topsoil | Temperate forest | FRB, FRN |
| Bronson et al. (2008) | +5 | Soil | Topsoil | Boreal forest | FRB |
| Wan et al. (2004) | +4 air, +1.2 soil | Air + Soil | Topsoil | Not specified | FRB, FRP, SRL |
| Dawes et al. (2015) | +4 | Soil | Topsoil | Temperate forest | FRB |
| Leppälammi-Kujansuu et al. (2013) | +5 | Soil | Topsoil & Subsoil | Boreal forest | FRB, SRL, RTD, AvgD |
| Parts et al. (2019) | +4 | Soil | Topsoil | Temperate forest | FRB, SRL, SRA, RTD |
| Björk et al. (2007) | +2–3 | Air | Topsoil | Dry tundra | SRL, SRA, RTD |
| Kwatcho Kengdo et al. (2022) and (2023) | +4 | Soil | Topsoil | Temperate forest | FRB, FRP, SRL, SRA, RTD, Mycorrhizal colonization |
| Solly et al. (2017) | +4 | Soil | Topsoil | Temperate forest | Mycorrhizal colonization |
| Heinzle et al. (2023) | +4 | Soil | Topsoil | Temperate forest | Root exudation |
| Liu et al. (2024) | +4 | Soil | Topsoil | Temperate forest | Root chemistry |
| Malhotra et al. (2020) | +0 °C, +2.25 °C, +4.5 °C, +6.75 °C, +9 °C | Air + Soil | Topsoil  Subsoil | Peatland | Fine root growth, root length |

References

Björk, R. G., Majdi, H., Klemedtsson, L., Lewis-Jonsson, L., & Molau, U. (2007). Long-term warming effects on root morphology, root mass distribution, and microbial activity in two dry tundra plant communities in northern Sweden. New Phytologist, 176(4), 862–873. https://doi.org/10.1111/j.1469-8137.2007.02231.x

Bronson, D. R., Gower, S. T., Tanner, M., & Linder, S. & Van Herk, I. (2008). Response of soil surface CO2 flux in a boreal forest to ecosystem warming. Global Change Biology, 14(4), 856–867. https://doi.org/10.1111/j.1365-2486.2007.01508.x

Dawes, M. A., Philipson, C. D., Fonti, P., Bebi, P., Hättenschwiler, S., Hagedorn, F., & Rixen, C. (2015). Soil warming and CO2 enrichment induce biomass shifts in alpine tree line vegetation. Global Change Biology, 21(5), 2005–2021. https://doi.org/10.1111/gcb.12819

Heinzle, J., Kitzler, B., Zechmeister-Boltenstern, S., Tian, Y., Kwatcho Kengdo, S., Wanek, W., Borken, W., & Schindlbacher, A. (2023). Soil CH4 and N2O response diminishes during decadal soil warming in a temperate mountain forest. Agricultural and Forest Meteorology, 329, 109287. https://doi.org/10.1016/j.agrformet.2022.109287

Kwatcho Kengdo, S., Ahrens, B., Tian, Y., Heinzle, J., Wanek, W., Schindlbacher, A., & Borken, W. (2023). Increase in carbon input by enhanced fine root turnover in a long-term warmed forest soil. The Science of the Total Environment, 158800. https://doi.org/10.1016/j.scitotenv.2022.158800

Kwatcho Kengdo, S., Peršoh, D., Schindlbacher, A., Heinzle, J., Tian, Y., Wanek, W., & Borken, W. (2022). Long-term soil warming alters fine root dynamics and morphology, and their ectomycorrhizal fungal community in a temperate forest soil. Global Change Biology, 28(10), 3441–3458. https://doi.org/10.1111/gcb.16155

Leppälammi-Kujansuu, J., Ostonen, I., Strömgren, M., Nilsson, L. O., Kleja, D. B., Sah, S. P., & Helmisaari, H.-S. (2013). Effects of long-term temperature and nutrient manipulation on Norway spruce fine roots and mycelia production. Plant and Soil, 366(1–2), 287–303. https://doi.org/10.1007/s11104-012-1431-0

Liu, X., Heinzle, J., Tian, Y., Salas, E., Kwatcho Kengdo, S., Borken, W., Schindlbacher, A., & Wanek, W. (2024). Long-term soil warming changes the profile of primary metabolites in fine roots of Norway spruce in a temperate montane forest. Plant, Cell & Environment, 47(11), 4212–4226. https://doi.org/10.1111/pce.15019

Malhotra, A., Brice, D. J., Childs, J., Graham, J. D., Hobbie, E. A., Vander Stel, H., Feron, S. C., Hanson, P. J., & Iversen, C. M. (2020). Peatland warming strongly increases fine-root growth. Proceedings of the National Academy of Sciences of the United States of America, 117(30), 17627–17634. https://doi.org/10.1073/pnas.2003361117

Melillo, J. M., Butler, S., Johnson, J., Mohan, J., Steudler, P., Lux, H., Burrows, E., Bowles, F., Smith, R., Scott, L., Vario, C., Hill, T., Burton, A., Zhou, Y.-M., & Tang, J. (2011). Soil warming, carbon-nitrogen interactions, and forest carbon budgets. Proceedings of the National Academy of Sciences of the United States of America, 108(23), 9508–9512. https://doi.org/10.1073/pnas.1018189108

Parts, K., Tedersoo, L., Schindlbacher, A., Sigurdsson, B., Leblans, N. I. W., Oddsdóttir, E. S., Borken, W., & Ostonen, I. (2019). Acclimation of Fine Root Systems to Soil Warming: Comparison of an Experimental Setup and a Natural Soil Temperature Gradient. Ecosystems, 22(3), 457–472. https://doi.org/10.1007/s10021-018-0280-y

Solly, E. F., Lindahl, B. D., Dawes, M. A., Peter, M., Souza, R. C., Rixen, C., & Hagedorn, F. (2017). Experimental soil warming shifts the fungal community composition at the alpine treeline. New Phytologist, 215(2), 766–778. https://doi.org/10.1111/nph.14603

Wan, Shiqiang., Norby, Richard. J., Pregitzer, Kurt. S., Ledford, Joanne., & O’Neill, E. G. (2004). CO2 enrichment and warming of the atmosphere enhance both productivity and mortality of maple tree fine roots. New Phytologist, 162(2), 437446. https://doi.org/10.1111/j.1469-8137.2004.01034.x

Zhou, Y., Tang, J., Melillo, J. M., Butler, S., & Mohan, J. E. (2011). Root standing crop and chemistry after six years of soil warming in a temperate forest. Tree Physiology, 31(7), 707–717. https://doi.org/10.1093/treephys/tpr066
